# Complete genome of *Variovorax* sp. EBFNA2, isolated from a surface-sterilized fava bean nodule

**DOI:** 10.1128/mra.00762-24

**Published:** 2024-11-11

**Authors:** Ann M. Hirsch, Ethan Humm, Liudmilla Rubbi, Giorgia Del Vecchio, Sung Min Ha, Matteo Pellegrini, Robert P. Gunsalus

**Affiliations:** 1 Department of Molecular, Cell, and Developmental Biology, University of California, Los Angeles, California, USA; 2 Department of Microbiology, Immunology, and Molecular Genetics, University of California, Los Angeles, California, USA; 3 Department of Integrative Biology and Physiology, University of California, Los Angeles, California, USA; 4 UCLA DOE Institute, University of California, Los Angeles, California, USA; California State University San Marcos, San Marcos, California, USA

**Keywords:** *Variovorax*, genomes, endophytes, PGPB

## Abstract

We report the complete genome sequence of *Variovorax* sp. EBFNA2, a fava bean nodule endophyte. The strain appears to be a new species, and its genome provides insight into its interactions with host plants and potential to promote plant growth and crop yield.

## ANNOUNCEMENT

In this study, the microbial community within a rhizosphere soil sample of wheat grown in Egypt (1) was used to induce nodules on fava beans from which various non-rhizobial endophytes were subsequently isolated. One of these, *Variovorax* sp. EBFNA2 (isolated 13 September 2018), is related to *Variovorax boronicumulans* (Table 1), which is known to accumulate boric acid intracellularly (2). Related strains are capable of degrading phenol (3), acrylamide (4), and insecticides (5, 6). Strain *V. boronicumulans* CGMCC 4969 was also shown to modulate the production of the phytohormone indole-3-acetic acid (IAA) and promote the growth of *Arabidopsis* (7).

**TABLE 1:** *Variovorax* sp. EBFNA2 genome information

Feature	Contig #1	Contig #2	Combined
Topology	Circular	Circular	Two circular
Size (bp)	6,463,879	938,298	7,402,177
GC%	67.5	65	67
Coverage (×)	37	37	37
Total raw reads	N/A^*a*^	N/A	180,945
Average read length	N/A	N/A	7,056.86
High-quality reads	N/A	N/A	24,166
*N*_50_ quality value	N/A	N/A	6,463,879
Completeness value	N/A	N/A	100%
Protein-coding genes	6,006	828	6,834
16S number	2	0	2
tRNA number	52	0	52
16S analysis^*b*^	N/A	N/A	99.93%
ANI analysis (%)^*c*^	94.03	77.92	93.94

^*a*^ N/A, not applicable.

^*b*^ Genomic 16S to *V. boronicumulans* BAM-48^T^.

^*c*^ Individual contig or whole genome compared to the whole genome of *V. boronicumulans* BAM-48^T^.

For strain isolation, fava beans were grown in 1-gallon pots containing sterilized potting mix, and nodulation was induced by mixing in wheat rhizosphere soil collected in Beheira Governorate, Egypt (30.904514, 29.878775) at 10 g soil per pot prior to sowing seeds. Plants were harvested 3 weeks post-germination, and surface-sterilized nodules were aseptically crushed with a mortar and pestle according to Youseif et al. (8). Serial dilutions were plated on LB agar and incubated at 30°C. Single colonies were picked and re-streaked to obtain pure cultures. DNA was extracted using the Quick-DNA HMW Magbead Kit (Zymo Research) and fragmented using Covaris gTubes (four passes at 7,000 rpm through the gTube orifice) following the manufacturer’s instructions. The average size of the sheared gDNA (8,036 bp) was checked at the TapeStation 4200 (Agilent). Multiplexed microbial libraries were prepared using the PacBio SMRTbell prep kit 3.0 together with the SMRTbell barcoded adapters 3.0 according to the PacBio protocol. Final whole genome libraries were not size-selected but simply purified via a standard procedure using 1× SMRTbell cleanup beads. DNA sequencing was performed using the PacBio Sequel IIe platform. Demultiplexing and adapter trimming were done using Lima version 2.9.0 (https://github.com/pacificbiosciences/barcoding). High-quality reads were assembled by Canu version 2.2 (9), and the assembled genomes were further refined by Circlator version 1.5.5 (10) to identify circular contigs, remove redundant non-circular contigs, and rotate circular contigs to start with *dnaA*. A completeness check was performed by CheckM version 1.0.18 (11), and the *N*_50_ quality was determined by Assembly stats version 1.01 (https://github.com/sanger-pathogens/assembly-stats). Genome ORF calling and annotation were performed by NCBI’s PGAP version 6.6 (12).

The genomic features of the two finished circular contigs are shown in Table 1. The ANI value against *V. boronicumulans* BAM-48^T^ (=NBRC 15149) (accession NZ_BCUS00000000.1) was 93.94%, suggesting that EBFNA2 represents a new species. ANI was calculated using contigs and the Ezbiocloud ANI Calculator (13). All software tools used default parameters that were stated in each tool’s manual.

The genome is enriched in genes related to heavy metal resistance (MerR family copper efflux transcriptional regulators, copper-responsive two-component system CusR/CusS, arsenate reductase, ArsH) and xenobiotic degradation (including genes related to degradation of chloroalkanes, dioxins, styrene, toluene, and xylene). Xenobiotic degradation/metabolism accounts for 3.92% of genes assigned to KEGG categories. Additionally, *V*. sp. EBFNA2 encodes the enzyme ACC deaminase (acdS), genes related to IAA biosynthesis, and acetoin and trehalose biosynthesis, all of which may contribute to plant growth promotion.

## Data Availability

Raw sequencing reads have been deposited under the SRA accession number SRR26382609, and the assembled genome is listed under the GenBank assembly accession number GCA_033802765.1.
