# Correction for Hirsch et al., “Complete genome of *Variovorax* sp. EBFNA2, isolated from a surface-sterilized fava bean nodule”

**DOI:** 10.1128/mra.01449-25

**Published:** 2026-02-12

**Authors:** Ann M. Hirsch, Sameh H. Youseif, Ethan Humm, Liudmilla Rubbi, Giorgia Del Vecchio, Sung Min Ha, Matteo Pellegrini, Robert P. Gunsalus

**Affiliations:** 1 Department of Molecular, Cell, and Developmental Biology, University of California, Los Angeles, California, USA; 2 School of Biotechnology, Nile University, Giza, Egypt; 3 Department of Microbial Genetic Resources, National Gene Bank (NGB), Agricultural Research Center (ARC), Giza, Egypt; 4 Department of Microbiology, Immunology, and Molecular Genetics, University of California, Los Angeles, California, USA; 5 Department of Integrative Biology and Physiology, University of California, Los Angeles, California, USA; 6 UCLA DOE Institute, University of California, Los Angeles, California, USA

## AUTHOR CORRECTION

Vol. 13, no. 12, e00762-24, 2024, https://doi.org/10.1128/mra.00762-24. The byline and affiliations should appear as given in this correction. Sameh H. Youseif was inadvertently omitted.

